# Lessons from failure: neurosurgical outreach in Managua, Nicaragua

**DOI:** 10.1007/s00381-021-05141-8

**Published:** 2021-08-24

**Authors:** Rahul Jandial, Pranay Narang, Jorge Daniel Brun Aramayo, Michael Levy

**Affiliations:** 1 Division of Neurosurgery, City of Hope National Medical Center, MOB 2001, 1500 East Duarte Road, Duarte, CA 91010 USA; 2 Halmos College of Natural Sciences and Oceanography, Nova Southeastern University, Fort Lauderdale, FL USA; 3 Hospital Del Nino, La Paz, Bolivia; 4 Division of Pediatric Neurosurgery, Rady Children’s Hospital-San Diego, Encinitas, CA USA

**Keywords:** Hydrocephalus, Neurosurgical outreach, Pediatric neurosurgery

## Abstract

With respect to the tremendous deficit in surgical care plaguing developing nations, it is critical that medical outreach models be organized in such a fashion that sustainable advancements can be durably imparted beyond the duration of targeted missions. Using a didactic framework focused on empowering host neurosurgeons with an enhanced surgical skillset, a mission was launched in Managua, Nicaragua, after previous success in Kiev, Ukraine, and Lima, Peru. Unfortunately, the failure to critically assess the internal and external state of affairs of the region’s medical center compromised the outreach mission. Herein lies the visiting team’s lessons from failure and insights on facilitating effective communication with host institutions, circumventing geopolitical instability, and utilizing digital collaboration and video-conferencing tools in the post-COVID-19 era to advance the surgical care of developing regions in a fashion that can be generationally felt.

## Introduction

With 1.7 billion children and adolescents lacking access to safe, timely, and affordable surgical care and anesthesia [2], various medical outreach models have been designed and tested in an effort to reduce the deficit in international surgical care. It is expected that the optimal framework for improving pediatric neurosurgical care internationally must be founded on a mission objective to teach neurosurgical skills such that operations can be routinely performed by local providers after visiting surgeons depart. By transferring operative skill, the collective surgical skill set of a region has potential to evolve over time. This didactic model can impart a much larger impact than medical outreach models where surgeons visit countries and solely provide direct care to citizens in need, an alternative that cannot be scaled or sustained beyond the duration of the mission. The latter model functions on a scheduled, elective basis and also cannot be utilized for pediatric neurosurgery, where care is usually provided on an emergent basis. The cost-effectiveness of perpetual, routine visits to provide care may also not be feasible for charitable entities with limited resources.

Recognizing the need for pediatric neurosurgical support in Managua, Nicaragua, a series of missions guided by this didactic model were launched in 2016. Given the high incidence of pediatric hydrocephalus, with patients receiving ineffective shunting operations that lead to shunt infection or malfunction, it was a clinical priority to displace the costly and risky ventriculoperitoneal shunting procedures with endoscopic operations. By teaching neuroendoscopic techniques, pediatric hydrocephalus could be more effectively resolved, and the newfound skills could also be applied to performing pediatric tumor biopsies, cyst fenestrations, and septum pellucidotomies. However, the outreach was ultimately disrupted during the second mission in 2018 as a result of the geopolitical tension of the time. While digital collaboration has been maintained, the outreach was left incomplete and operative skill has not been sufficiently transferred. With the COVID-19 pandemic having jeopardized the future outlook on supporting Managua, it should be seen that the time, capital, and resources that were invested in planning and executing the neurosurgical outreach could have been better utilized if applied elsewhere.

Providing international surgical support plays a large tax on the limited resources that charitable organizations and academic entities have. The intent of this report is to elucidate the challenges faced and lessons learned from the failed pediatric neurosurgical outreach in Managua to ensure that future endeavors focused on providing international surgical support can be successfully carried out, even in similar regions where the political climate may be challenging to navigate.

## Mission objective

The intent of the mission was to empower host pediatric neurosurgeons with an improved neurosurgical skill set that could be utilized beyond the duration of the visit to serve citizens in need. It became apparent that there was a high incidence of hydrocephalus among children, with causes including tumors, head injuries, meningitis, hemorrhage, and defects since birth. The standard of care involved ventriculoperitoneal shunting procedures, which are time-consuming and costly and carry a risk of infection and shunt malfunctioning. Consequently, the outreach in Managua was focused on transferring neuroendoscopy operative skills to the host neurosurgeons to treat such cases more effectively.

## Timeline and mission progress

After establishing digital contact in 2015, it was noted that the faculty of the Hospital Escuela Antonio Lenin Fonseca, an institution in the capital of Nicaragua which supports the only neurosurgery residency program in the country, were in need of neurosurgical support. In 2016, the visiting team conducted an in-person site evaluation, verifying the need for a robust pediatric neurosurgery outreach through which surgical support and equipment could be offered and operative skill transferred to the faculty. The program could benefit from improved organization of the existing resources, improved tracking of patients and their long-term outcomes, and enhancements of the providers’ surgical skills.

In 2017, the first 1-week mission was conducted during which the necessary foundation was established. In 2018, however, during the second 1-week mission, the geopolitical circumstances of the region disrupted the mission, bringing the effort to an immediate close. The third mission, in light of the continued geopolitical challenges and the recent COVID-19 pandemic, has also not been executed.

During the first mission, 6 neuroendoscopic cases took place to treat hydrocephalus, and 5 general neurosurgical cases were performed on children. In terms of the utility of the partial outreach, it could be said that the faculty’s capacity to carry out the logistics of neurosurgical care have been enhanced.

## Lesson 1: Effective communication with hospital leadership/administration is just as important as communication with host faculty

The first challenge of an international medical initiative is to ensure that both the host neurosurgical faculty *and the hospital leadership* are devoted to enhancing the surgical capacity of the institution. Since there are countless communities in need of surgical support, the dedication of the faculty and administration is necessary to ensure that the impact can be scaled over time. A dedication to improving the welfare of the indigent citizens was verified during the site evaluation in 2016. It was also apparent that the faculty were capable of carrying out the basic logistics for neurosurgical care, including patient selection, operative schedule management, nursing and beds management, and post-operative care. Initially, the administration and hospital leadership stood by the mission objective and ensured that other surgeons would relinquish their operating days and that nurses would be prepared to manage post-operative needs during the missions. *While administrative support was present during the initial site evaluation and during the first mission in 2017, the new appointment of hospital leadership in 2018 did not demonstrate this support.*

The visiting team and the host neurosurgical faculty were in close communication in the months leading up to the second mission, but with the new leadership disconnected from the past mission, their understanding of the neurosurgical outreach was fragmented at best and ultimately translated to a disinterest in the missions.

It had been challenging to maintain communication with the new leadership, but communication was actively in place with the host neurosurgeons. From speaking with the surgical faculty, the team was assured that even though the region could be characterized by tremendous political instability in the months preceding the visit, they would be able to travel and ultimately execute their mission, overcoming any international obstacles that would arise in the process. However, it became apparent far too late that the word of the neurosurgical faculty was not enough. Upon arriving at the institution at the beginning of the second mission, the administration and hospital leadership informed the visiting team that they did not have permission to operate or teach. The leadership immediately redirected the team to the airport to leave the country, partly out of fear that a geopolitical crisis could ensue.

*Future missions should seek to ensure that if there is an expected change in hospital leadership over the course of the outreach, the newly appointed members, as well as the host neurosurgical faculty, are committed to the mission. Communication with administration is of paramount importance: without administrative support, it is questionable whether the resources are being allocated most appropriately.*

## Lesson 2: Geopolitical instability—an overlooked factor that should be thoroughly assessed

The second challenge is designing and executing an instructive, multilayered system through which operative skills can be transferred in such a fashion that they are not lost over time. As with the previous missions, the system that was implemented in Managua included a series of three 1-week missions. The first mission would establish a foundation for future missions, forging relationships with the faculty and cultivating an engaging learning environment. During this first mission, the visiting team would lead the surgical care for neuroendoscopy cases, carrying out operations interspersed with grand-round style case presentation and discussion while allowing the host surgeons to serve as assistant surgeons during procedures. The week should also begin with a joint clinic where pediatric patients can be evaluated together, allowing both the visiting and host team to mutually decide the operations that will be performed. The second mission would be more focused on the didactic elements, with the surgeries being performed more collaboratively: the visiting team would serve as the primary surgeons and the host team would take on increased responsibility as the assisting surgeons, with some host surgeons taking a primary role based on their skill level. In the third mission, the host surgeons would take on even more responsibility, serving as the primary and assisting surgeons for all cases, with the visiting team playing an observational role, providing constructive support to refine the skill set of the host surgeons. By spreading these missions over a 3-year span, the host neurosurgeons would be gradually empowered to take on increased responsibility over time. This system ensures that the lessons were retained, mastered, and extended over the course of the 3-year outreach. *While the first mission in 2017 was effective at laying the foundation, this system was shattered when the political climate in 2018 resulted in abrupt closure of the second mission.*

In April of 2018, President Ortega and Vice President Murillo ordered police and parapolice forces to violently bring an end to the peaceful protests that had been catalyzed over discontent with a government decision to reduce social security benefits. The government’s response involved live ammunition and snipers, and as of November of 2018, the conflict left 325 people dead, more than 2000 people injured, hundreds illegally detained and tortured, and 52,000 exiled in neighboring countries. Human rights were markedly deteriorated, with issues including reports of unlawful and arbitrary killings of civilians; force disappearance by parapolice forces; torture; human trafficking; attacks against lesbian, gay, bisexual, transgender, and intersex (LGBTI) persons; physical abuse; rape by government officials; arbitrary arrest and detention; harsh and life-threatening prison conditions; arrests of journalists; censorship; criminal libel; and substantial interference with the rights of peaceful assembly and freedom of association, including attacks on the Roman Catholic Church and Church officials. The protests are currently ongoing and have been since April of 2018 [3].

With the civil unrest and political tension permeating the air, events in the days prior to the second mission had stricken fear among the hospital leadership that a protest might ensue. *The host neurosurgical faculty did not share this sentiment, and the lack of communication with the hospital leadership resulted in the visiting team travelling to Managua, only to be escorted back to the airport by officials to leave the country.*

The magnitude of the geopolitical instability was overlooked in 2018, and the investments that had been made in terms of capital, resources, and time could have been more optimally allocated had the outreach taken place in a different country in need of support. *It is the authors’ opinions that when selecting a site for a medical initiative, extensive communication with local citizens, a political expert of the region in the USA, and an official in the host country should take place.*

## Lesson 3: The utility of digital collaboration and video-conferencing tools

The third lesson that was derived from the failed outreach was the importance of maintaining communication digitally. After the abrupt dismissal from Managua, Nicaragua, the visiting team maintained contact digitally throughout 2018 and 2019. Though the neuroendoscopic operative skill transfer was left incomplete, the lessons surrounding the logistics of neurosurgical care and post-operative management that were taught during the first were reinforced through video-conferencing services. During the COVID-19 pandemic, communication increased and constructive feedback was provided to improve the resource utilization and patient capacity of the institution. The hospital had been overwhelmed with COVID-19 efforts, and with all resources dedicated to the deluge of cases, neurosurgical work had been deferred indefinitely. The visiting team invited the host neurosurgical faculty to virtual biweekly morbidity and mortality conferences, and virtual events where neurosurgeons, residents, post-doctoral fellows, and fourth year medical students presented and listened to an array of neurosurgical topics. Aside from the academic alliance, the visiting team also connected their hospital leadership with the hospital leadership of Hospital Escuela Antonio Lenin Fonseca, fostering conversations that would ultimately advance the rate at which COVID-19 treatment protocols in the USA were adopted by the institution.

It is expected that following the pandemic and the geopolitical tension that is still present today, a neurosurgical outreach in Managua will be replicated, likely using digital video-conferencing services such as Zoom to a greater degree. The hyper digital model used to discuss educational topics between neurosurgical faculty at Johns Hopkins through the pandemic [1] can be replicated and scaled to provide host neurosurgeons in Managua with learning opportunities, supplementing an in-person didactic effort where newfound surgical skill sets can be acquired.

## Acting in ignorance: thoughts for future medical endeavors

Reflecting on the failed second mission in Managua, Nicaragua, it is clear that a number of mistakes were made naively, each of which was preventable.

Fundamentally, international medical missions are founded on the assumption that doctors visiting from first world countries carry a skill set that is difficult to match in developing nations.

While it may be favorable for international medical centers to collaborate with such doctors, the international obstacles in place today will not dissipate in the face of any such opportunity.

The fact that the geopolitical obstacles of the region were overlooked was a serious lapse in judgement. Relationships between the USA and South America were strained, and extensive communication with administrative members and leadership should have been a prerequisite for any consideration of a second mission. Such communication would have clarified the new position of the institution on this medical outreach. The resources, time, and attention that were devoted to the endeavor could have been allocated to a different developing nation, and an opportunity to impart a tangible impact on pediatric neurosurgical care in a sustainable fashion could have been brought to fruition.

Future international pediatric neurosurgical endeavors should seek to first forge relationships with different levels of hospital administration, hospital faculty, and hospital leadership. Once it becomes clear that a commitment to advancing the quality of pediatric neurosurgical care is apparent at each tier of the institution, a strategic design founded on teaching operative technique (instead of solely providing care) should be proposed, addressing the specific needs of the center in consideration. Prior to the inaugural mission, there should also be a thorough assessment of the political stability of the region, with inputs taken into account from both the host country’s political officials as well as US officials. Given that certain teams—in our case, the neurosurgical faculty—may have different opinions on the volatility of the region and the risk of visiting, a simple rule should be followed: if there is any founded concern that geopolitical stability could threaten completion of the outreach, the missions should be deferred or launched elsewhere. These are foundational requirements to substantially impact the neurosurgical care of a region. Any deviation from these components, as illustrated by the failed effort in Managua, Nicaragua, will translate to resources dedicated suboptimally, and an impact that will collapse on itself soon after completion of the missions.

## Discussion

Neuroendoscopic techniques, when effectively transferred, can serve in a multifaceted fashion, restoring the lives of children suffering from hydrocephalus, while providing diagnostic opportunities (e.g., tumor biopsies) that can catch abnormalities prior to the onset of disease. Still, enhancing the neurosurgical capacity of a facility is a challenging effort that requires a rigorous mission design that can sustain the impact of the endeavor beyond the missions. From analyzing the causes for the failed pediatric outreach in Managua, Nicaragua, it can be concluded that future medical outreach missions should critically assess the internal and external state of affairs of a medical center before devoting resources to it.

Internally, it is necessary to have the support of the hospital leadership prior to, during, and following completion of the medical outreach as their support will ensure that the newly acquired techniques are practiced, mastered, extended, and passed onto future generations of physicians. Without this support, the impact cannot be reliably maintained and will be episodic and short termed in nature. Externally, geopolitical stability is a critical requirement so that the planned medical outreach model can be wholly executed, without interference or hindrance during the missions or afterwards.

While it is difficult to predict changes in geopolitical stability and hospital leadership, this challenge can be navigated by conversing with political experts as well as the local citizens, hospital faculty, personnel, and present leadership. If there is doubt that the internal or external state of affairs are subject to change, one should be reluctant to execute the medical endeavor in that region, as there is risk that the outreach model will be disrupted. It should also be seen that with the cultural change that has taken place globally as a result of the COVID-19 pandemic, with increased utilization of telehealth services and video-conferencing tools, there is potential for a digital education model to be prepared as a means of improving the quality of pediatric neurosurgical care delivered globally. These tools can also preserve the impact that a medical outreach mission imprint and can be employed to reinforce lessons and improve management of the logistics surrounding pediatric care. Future missions should take these components into account when selecting a region to provide surgical support in, especially for emergent specialties such as pediatric neurosurgery.
